# Feeling versus understanding: lived-experience insights into perceived mechanisms of change of creative arts and psychomotor therapies versus verbal therapeutic approaches

**DOI:** 10.3389/fpsyt.2026.1863648

**Published:** 2026-08-27

**Authors:** Jackie Heijman, Karin Timmerman, Imke Wiersma, Hans Wouters, Mirjam Radstaak, Gerben Westerhof, Dwayne Meijnckens, Suzanne Haeyen

**Affiliations:** 1 Research Group Arts and Psychomotor Therapies in Health Care, Academy of Health and Vitality, HAN University of Applied Sciences, Nijmegen, Netherlands; 2 Specialist Centre for People with Personality Disorders, De Boerhaven, Mediant, Hengelo, Netherlands; 3 Psychology, Health & Technology, Faculty of Behavioral, Management and Social Sciences (BMS), University of Twente, Enschede, Netherlands; 4 Specialist Centre for People with Personality Disorders, Scelta, GGNet, Twente, Netherlands; 5 Department of Knowledge, Innovation and Research, MIND, Amersfoort, Netherlands; 6 Specialist Centre for People with Personality Disorders, Scelta, GGNet, Nijmegen, Netherlands

**Keywords:** client perspective, complementary therapies, creative arts and psychomotor therapies, embodiment, emotion regulation, lived experience, psychotherapy, shared decision making

## Abstract

**Introduction:**

In mental health care, there is growing interest in embodied and experiential approaches such as creative arts and psychomotor therapies (CAPTs), which actively engage emotions and bodily experience in treatment. At the same time, service users’ perspectives are increasingly recognized as an important source of knowledge within evidence-based practice. This study investigated how people with lived experience perceive CAPTs in relation to verbal therapeutic approaches.

**Methods:**

A qualitative survey among people with lived experience (n = 543) examined perceived helpfulness of CAPTs and verbal approaches, as well as experienced differences between them.

**Results:**

Findings indicate that CAPTs and verbal therapies are viewed as complementary. CAPTs are described as active and experiential, particularly helpful for connecting with emotions and increasing bodily awareness, whereas verbal therapies are seen as primarily verbal and cognitive, especially helpful for gaining insight into problems and fostering a sense of being heard and understood.

**Discussion:**

These results underline the importance of integrating emotional and bodily awareness into therapy and suggest that combining embodied and verbal approaches may address complementary aspects of therapeutic change for people with mental health difficulties.

## 1 Introduction

According to the World Health Organization (WHO) (1), nearly one billion people worldwide have mental health problems. This equates with approximately one in eight people. Mental health care offers a wide range of treatments for mental health problems, including both verbal therapeutic approaches and creative arts and psychomotor therapies (CAPTs).

Verbal therapeutic approaches focus primarily on addressing mental health problems through structured conversation (2). These approaches are supported by substantial empirical evidence and are considered first-line treatments for many DSM-5-TR diagnoses. At the same time, treatment outcomes vary across individuals, underscoring the importance of considering differences in needs, preferences, and clinical presentations (3). For example, meta-analyses indicate that cognitive behavioral therapy (CBT) is highly effective for anxiety disorders, while outcomes for other conditions, such as depression, bipolar disorder, or personality disorders, are more variable (3, 4). This variability highlights the value of a diverse therapeutic landscape in mental health care.

CAPTs represent a group of therapeutic approaches in which experiencing, doing, and embodied engagement form the therapeutic starting point (5). These therapies include visual art, dance, drama, music, play, and psychomotor therapy. CAPTs employ creative and body-based modalities to support mental and emotional well-being by addressing psychological, developmental, and social challenges (6, 7). Conceptually, they are often described as primarily bottom-up approaches, emphasizing sensory and embodied experience, whereas verbal therapeutic approaches are typically characterized as more top-down, starting from cognition and language (8). CAPTs are delivered by accredited and qualified therapists and may be particularly relevant when experiential or non-verbal processes play a central role in treatment.

**Figure 1:**
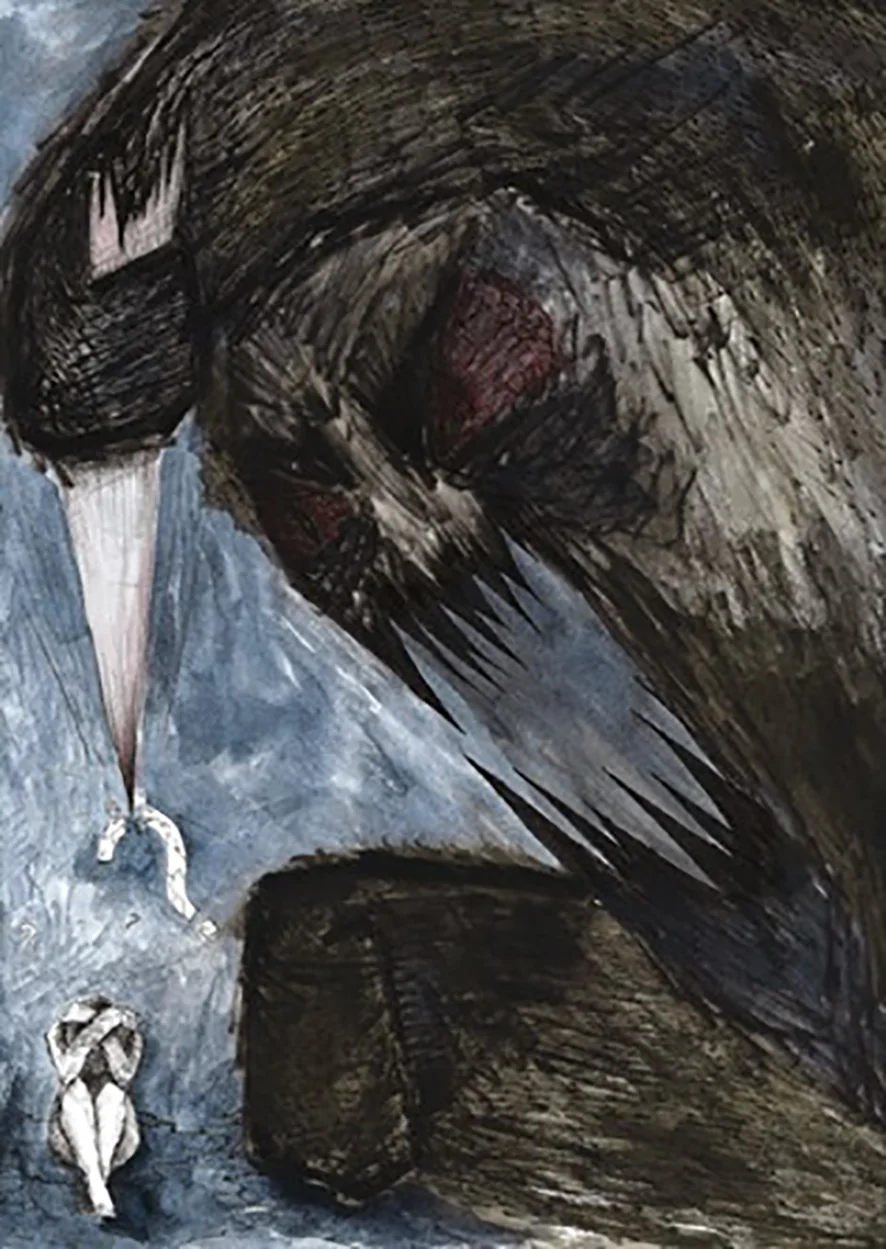
Visualization of fear in art therapy. Created by a patient and published with consent.

Examples of CAPTs-interventions include visualizing emotions (**Figure 1**), or enacting conflict scenes in dramatherapy to practice assertive behavior. Such interventions illustrate several proposed active factors of CAPTs, including embodiment (engaging bodily experience in the therapeutic process), concretization (making abstract experiences tangible through materials or movement), and symbolism/metaphor (expressing complex emotions or relational patterns symbolically) (5). Through these processes, experiences that may be difficult to articulate verbally can be explored in an experiential and structured manner. Evidence for CAPTs is growing, with promising results for, for example: individuals with personality disorders (9, 10), anxiety and stress (11, 12), pain in palliative care (13), and children with poor social-emotional skills (14, 15). Recent studies also examine the mechanisms and active factors underlying these therapies: how they work (5, 16). Despite this growing evidence base, further research is needed to deepen and broaden the scientific understanding of CAPTs.

While quantitative studies increasingly examine outcomes and mechanisms of CAPTs, understanding how these therapies are experienced in practice remains equally important. When multiple therapeutic approaches are available, insight into patients’ lived experiences through qualitative research is essential for informing evidence-based practice and clinical decision-making (17). Evidence-based practice (EBP) traditionally rests on three pillars: the best available research evidence, clinical expertise, and patient preferences and values (18). However, patients’ lived experiences, despite being a core component of high-quality care, are often underrepresented in the scientific literature (19). Qualitative research addresses this gap by foregrounding participants’ perspectives, meanings, and contextualized understandings in ways that quantitative measures cannot, thereby translating experiential knowledge into meaningful evidence within EBP (17). Incorporating experiential knowledge helps ensure that treatment is not only empirically supported but also aligned with individuals’ needs, preferences, and personal contexts.

This study is therefore relevant to the principle of shared decision making, in which clinicians and patients collaboratively determine the most appropriate treatment option. Taking patients’ preferences and experiences into account has been associated with lower dropout rates and improved treatment outcomes (20, 21). In contexts where both verbal therapeutic approaches and CAPTs are available, understanding how individuals experience these modalities is therefore crucial.

The aim of this study is to examine how people with lived experience perceive and describe CAPTs, with particular attention to the working mechanisms they associate with these interventions. In addition, the study explores how these perceived mechanisms and experiences compare to those attributed to verbal therapeutic approaches.

## 2 Materials and methods

### 2.1 Design

This study used a qualitative survey to explore the experiences of people with lived experience with CAPTs and verbal therapeutic approaches.

### 2.2 Participants

Participants were recruited through MIND Platform. MIND Platform is the national umbrella organization of, for, and by client and family organizations in the mental health sector. MIND aims to systematically improve the situation of everyone affected by mental health issues and serves as a dialogue partner and point of contact for politicians, policymakers, policy implementers, health insurers, and industry organizations. MIND has a mental health care panel that provides feedback on current themes in health care, members of which receive 8–10 survey invitations per year via email and participate voluntarily. For the current study, panel members (approximately 4, 000) were invited to participate in a survey about CAPTs and wellbeing, that was open from May to June 2023. Eligibility required panel membership and provision of informed consent. In total, 543 individuals responded (see **Table 1** for participant characteristics). Among these participants, most identified as female (83%). The mean age was 49.06 years (SD = 13.44). Most had completed higher professional education (HBO), and the majority (93.2%) had self-related experience in mental health care. Of all participants, 63% reported experience with CAPTs. Among these participants, psychomotor therapy and art therapy were the most well-known CAPTs, while dance therapy and play therapy were least familiar. Drama therapy and music therapy fell in the mid-range of familiarity.

**Table 1:** Participant characteristics and familiarity with CAPTs (N = 543).

Variable	n (%)
Gender	
Male	87 (16%)
Female	448 (83%)
Other	8 (1%)
Education level	
Primary school	5 (1%)
Lower vocational education	20 (4%)
MULO, MAVO	36 (7%)
Higher general education (HAVO)	26 (5%)
Pre-university education (VWO)	26 (5%)
Higher professional education (HBO)	185 (34%)
University	105 (19%)
Lived experience	
Personal lived experience (self-identified)	506 (93.2%)
Familial experience	37 (6.8%)
Experience with CAPTs	
Yes No	342 (63%) 201 (37%)
Familiarity with CAPTs*	
Psychomotor therapy	284 (83%)
Art therapy	277 (81%)
Music therapy	133 (39%)
Drama therapy	130 (38%)
Play therapy	65 (19%)
Dance therapy	51 (15%)

CAPTs, creative arts and psychomotor therapies. *Percentages for familiarity are based on participants reporting experience with CAPTs. Multiple answers could be given.

### 2.3 Measures

The online survey was co-developed by the research team and a MIND Platform researcher who is also an expert by experience. The survey was originally created to support the development of a CAPTs treatment protocol for patients with a personality disorder for a separate study (22), but proved to serve broader research regarding perspectives of people with lived experience in mental healthcare.

The complete survey consisted of 21 questions-, including demographics, perceived helpfulness and unhelpfulness of CAPTs, perceived helpfulness and unhelpfulness of verbal therapeutic approaches, the difference between CAPTs and verbal therapeutic approaches, and treatment goals to be incorporated into therapies. Question formats included five open questions (such as: “In what way have CAPTs helped you?”), and several multiple-choice questions (e.g.: “which themes do you find most important to incorporate in therapy”) (see **Supplementary Material** for full survey). We anticipated that the survey would take approximately 10–15 minutes to complete but it had no time constraint. It was administered digitally using Spidox 2.0. A total of 486 participants completed the open-ended questions, of whom 330 (68%) had experience with CAPTs. In this study, the questions about patient characteristics, therapy priorities and open-ended questions were analyzed and reported. Questions specific to people with personality disorders and statements about art therapy were omitted (they were not comparable with verbal therapeutic approaches).

### 2.4 Procedure

A contract regarding data management and exchange was signed between HAN University of Applied Science and MIND mental health panel. An expert by experience from MIND provided feedback throughout the study (DM). Ethical approval was obtained from the Ethics Committee of the HAN University of Applied Sciences (ref: ECO 471.07/23). The study was registered as part of a larger study at https://www.ClinicalTrials.gov (trial registration number NCT06219122).

Participants received an email with a survey link and completed informed consent before beginning the questionnaire. The survey was administered once, and responses were anonymous. Some questions, regarding CAPTs, were only answered by those who had experience with CAPTs. Participants without this experience were guided to questions on verbal therapeutic approaches.

### 2.5 Analysis

#### 2.5.1 Descriptives and open-ended questions

Descriptive statistics were calculated for demographic and background variables (means and standard deviations for continuous variables, frequencies, and percentages for categorical variables). Analyses were conducted in SPSS 30.

#### 2.5.2 Qualitative analysis

Qualitative data consisted of open-ended responses about participants’ experiences with CAPTs and verbal therapeutic approaches. Analysis followed an inductive thematic approach (23) using ATLAS.ti 23.3. Three CAPTs researchers (two art therapists and one music therapist) independently coded the same subset of responses to ensure analytic rigor. After initial independent coding, the researchers (CAPTs therapists and a general psychologist) held meetings to compare interpretations, discuss discrepancies, and refine the coding schemes. This was meant for purpose of reflexivity. This process continued until consensus was reached, and a stable set of codes was established.

After agreement on the coding structure, one researcher (JH) grouped codes into overarching themes, consulting the other two researchers (IW and KT) at three stages to maintain consistency and reduce researcher bias. This form of investigator triangulation strengthens credibility and trustworthiness by incorporating multiple perspectives in the interpretive process (24). Themes were reported in sequence of most- to least mentioned themes. Representative quotes were selected to illustrate key themes and patterns identified during the analysis. Quotes were chosen based on their clarity, relevance to the theme, and their ability to reflect commonly expressed participant perspectives while preserving variation across participants.

#### 2.5.3 Linguistic inquiry and word count

We performed a Linguistic Inquiry and Word Count (LIWC), using the Dutch edition (25). With LIWC, one can count different categories of words from datasets, giving a quantitative presentation of narrative data. Relevant categories that could be matched to themes of the thematic analysis were selected. Word counts were compared on the following categories: affect; cognitive processes and its subcategory insight; perception and its subcategory feel; and body. The data were prepared for SPSS analyses. Means and standard deviations were computed and paired sample T-tests and non-parametric Wilcoxon signed-ranked tests were performed, because of skewed data, comparing word use in responses to the (un-)helpfulness of CAPTs with word use in response to the (un-)helpfulness of verbal therapeutic approaches. Alpha was set at.05. When participants chose not to answer a question, numbers for the LIWC were set on 0. Since it is possible that participants answered questions about verbal therapeutic approaches but not about CAPTs, and vice versa, the valid number of respondents (listwise) for the comparisons includes the group that answered both categories.

## 3 Results

### 3.1 Participant characteristics and therapy priorities

Participants rated their quality of life as suboptimal (M = 4.3 out of 7). Sixty-five percent were momentarily treated for psychological symptoms. Most experienced symptoms in this moment or in the past consisted of somberness (69%), negative self-image (56%), anxiety (55%), physical symptoms (50%), trauma symptoms (42%), and expression and regulation of emotions (35%).

From a list of life challenges and therapeutic themes, participants selected five they considered most important to address in therapy (**Table 2**). Notably, the most frequently selected theme was “being able to feel signals from your own body.” Other commonly chosen priorities included “coping with your own emotions, ” “processing the past, ” “gaining insight into your own behavior, ” and “recognizing your emotions.”

**Table 2:** Survey results on desired themes incorporated into therapy (n = 539).

Outcome	Frequency	% of total
Being able to feel signals from your body	255	47%
Coping with your emotions	217	40%
Processing the past	200	37%
Gaining insight into your own behavior	186	34%
Recognizing your emotions	167	31%
Adopting healthy ways of relating to yourself	162	30%
Changing (negative) thoughts	152	28%
Fulfilling your own needs in a healthy way	140	26%
Gaining new, positive experiences	134	24%
Reducing symptoms	127	23%
Knowing who you are	112	20%
Finding balance between your own needs and those of others	107	20%
Having fun	108	20%
Reinforcing your personal strengths	104	19%
Learning to adapt to changing circumstances	80	15%
Speaking to yourself in a compassionate/helpful way	78	14%
Knowing your own needs	66	12%
Developing new skills	66	12%
Adopting healthy ways of relating to others	57	10%
Knowing your strengths	57	10%
Better understanding the behavior of others	39	7%
Knowing the needs of others	3	1%

Percentages may sum to more than 100% because participants were allowed to select multiple options. *n* = 539; eight participants skipped this question.

### 3.2 (Un-)helpfulness of CAPTs and verbal therapeutic approaches

Open coding yielded 1, 564 initial codes, which were reduced to 933 after merging duplicates and synonyms. Through an iterative selective coding process with three researchers, these were further refined into 156 main codes. These main codes were then organized into five overarching categories, 37 main themes and 94 sub-themes. The five categories aligned with the content of the five open-ended questions, supplemented by one additional “other/remaining” category which showed general therapy conditions (see **Supplementary Material**).

#### 3.2.1 Category 1: helpfulness of CAPTs

**Figure 2:**
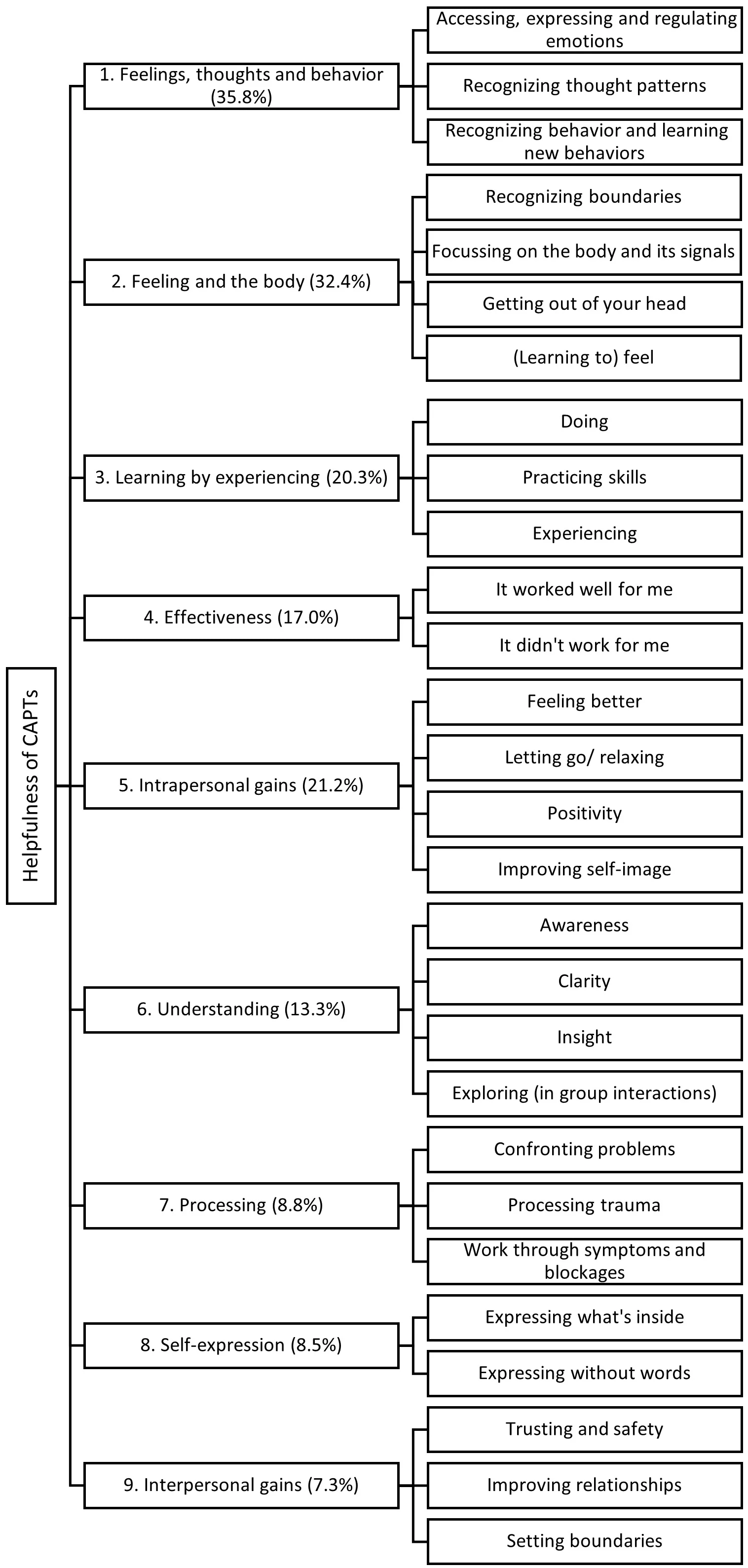
Code tree helpfulness of CAPTs. *N_CAPTs_=330*.

**Figure 2** shows a code tree with the main themes and corresponding sub-themes following the answers of the participants who experienced CAPTs to the question: in what way have CAPTs helped you? (*n* = 330).

##### 3.2.1.1 Theme 1: feelings, thoughts and behavior (35.8%)

The largest theme following the answers of the participants on helpfulness of CAPTs was that they experienced CAPTs as valuable for improving contact with feelings, thoughts and behavior (35.8%). With great emphasis on feeling, helping them recognize, allow, and express feelings beyond verbal expression. About 29% mentioned emotional gains, including accessing emotions, expressing them, identifying them, and depicting emotions visually or physically. For instance, one participant noted, *“It helped me to regulate my emotions, to sense them in my body, and to respond to them in a healthier way”.* Participants were also able to recognize thought- and behavioral patterns.

##### 3.2.1.2 Theme 2: feeling and the body (32.4%)

Another related strong theme was feeling/sensing the body (32.4%). 12% explicitly highlighted the mechanism of moving “out of the head” into physical awareness in CAPTs: *“By working with movement, I dared to be more present in my body and less in my thoughts”.* Participants also noted recognizing bodily signals and learning to feel, while 9% mentioned greater boundary awareness. “*In CAPTs I experienced that I always cross my boundaries, and I could practice what would happen when I don’t”.* In this way, patients mentioned that CAPTs supported embodiment, self-regulation, and reductions in physical complaints.

##### 3.2.1.3 Theme 3: learning by experience (20.3%)

The mechanism of experiential learning (20.3%) and related behavioral change were closely connected: participants described that actually *doing*, practicing new behaviors, coping strategies, and social skills in a safe setting within CAPTs. As one participant said, *“By seeing that I could already apply new behavior in the therapy setting, it became easier in the real world”.* CAPTs encouraged active engagement and learning through experiencing, according to participants.

##### 3.2.1.4 Theme 4: effectiveness (17.0%)

Overall, unprompted, 9.4% reported that CAPTs helped them without mentioning a specific related theme, while 7.6% indicated they were not helpful.

##### 3.2.1.5 Theme 5: intrapersonal gains (21.2%)

Participants also described intrapersonal gains (21.2%), such as feeling better, being able to relax more and let go of perfectionism, feeling more positive, or improving self-image: *“It gave me a stronger feeling of who I am and what I am worth”.*

##### 3.2.1.6 Theme 6: understanding (13.3%)

Understanding (13.3%) was another reported benefit, with participants gaining awareness, clarity and insight through CAPTs into emotions, behavior, and relational patterns, particularly through exploring in group interactions.

##### 3.2.1.7 Theme 7: processing (8.8%)

In addition, processing (8.8%) was noted, including confronting problems and processing trauma: *“I had processed many things cognitively, but not truly. My trauma was stored in my body, and for a long time I couldn’t access it. Through creative arts psychotherapy, I was finally able to truly process it and work on my recovery.”* Participants hereby noted working *through* blockages and symptoms.

##### 3.2.1.8 Theme 8: self-expression (8.5%)

Self-expression (8.5%) was described as helpful, meaning CAPTs enabling expression of inner experiences that were difficult to put into words: *“By painting or moving, I could show and feel that I was angry or sad”.*

##### 3.2.1.9 Theme 9: interpersonal gains (7.3%)

Finally, participants also described interpersonal benefits (7.3%), including greater safety and trust, improved relationships, and greater ability to set boundaries: *“It helped me to tolerate proximity, and to sense and set boundaries”*.

#### 3.2.2 Category 2: unhelpfulness of CAPTs

**Figure 3:**
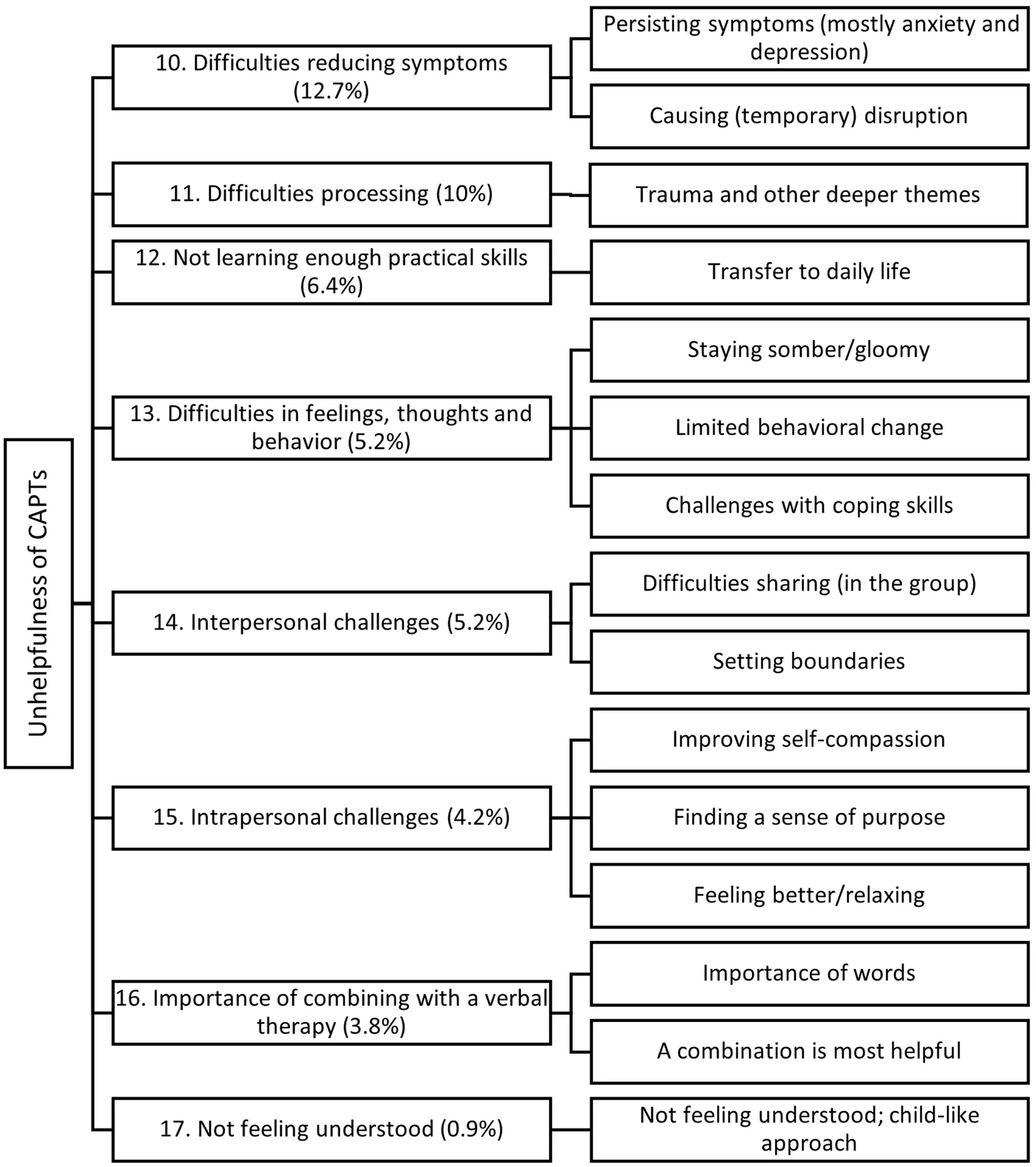
Code tree unhelpfulness of CAPTs. *N_CAPTs_=330*.

**Figure 3** shows a code tree with the main themes and corresponding sub-themes following the answers of the participants who experienced CAPTs (n=330) on what they did not experience as helpful about CAPTs.

##### 3.2.2.1 Theme 10: difficulties reducing symptoms (12.7%)

CAPTs did not always help to reduce symptoms (12.7%) according to participants. Some participants still had persisting depressive episodes or anxious feelings: *“Eventually, the depression keeps coming back”.* Others noted that CAPTs gave them more insight, but this could also be dis-regulative: participants were more aware of their themes but were not (yet) capable of working through them.

##### 3.2.2.2 Theme 11: difficulties processing (10%)

Even though 8.8% had mentioned helpfulness of CAPTs in processing trauma, other participants also mentioned that trauma and deeper themes were difficult for them to process within CAPTs (10%).

##### 3.2.2.3 Theme 12: not learning enough practical skills (6.4%)

Some participants mentioned that they were note learning enough practical skills to be able to handle daily life (6.4%).

##### 3.2.2.4 Theme 13: difficulties in feelings, thoughts, behavior (5.2%)

Regarding feelings, thoughts, and behavior (5.2%), some participants mentioned that CAPTs couldn’t help to take away gloomy mood. Others mentioned that CAPTs were helpful in the moment, but that it was difficult to really change coping and behavioral patterns and to transfer what they learned into daily life *“Truly changing behavior is difficult”*.

##### 3.2.2.5 Theme 14 interpersonal challenges(5.2%)

Interpersonal challenges (5.2%) included feeling unsafe in groups or difficulty sharing and working on boundaries. “*I bottled up my feelings even more in a group. I didn’t dare show my true self because I felt unsafe”.*

##### 3.2.2.6 Theme 15: intrapersonal challenges (4.2%)

Intrapersonal (4.2%) benefits were limited within CAPTs for some: 10 participants (3.0%) mentioned they did not feel more self-compassion or gain a better self-image, and four participants (1.2%) did not feel better or gain meaning in life through CAPTs. *“With art therapy, I am too critical of the result. Others can always do it better or differently than I can”.*

##### 3.2.2.7 Theme 16: importance of combining with a verbal therapy (3.9%)

3.9% emphasized the importance of combining CAPTs with verbal therapeutic approaches, noting that CAPTs alone did not help put experiences into words, which can be important. One participant stated, *“For me, the combination of verbal therapeutic approaches and art therapy made all the difference. Both were crucial”.*

##### 3.2.2.8 Theme 17: not feeling understood (0.9%)

3 participants (0.9%) mentioned that they did not feel understood in CAPTs; they experienced the approach as too child-like.

#### 3.2.3 Category 3: helpfulness of verbal therapeutic approaches

**Figure 4:**
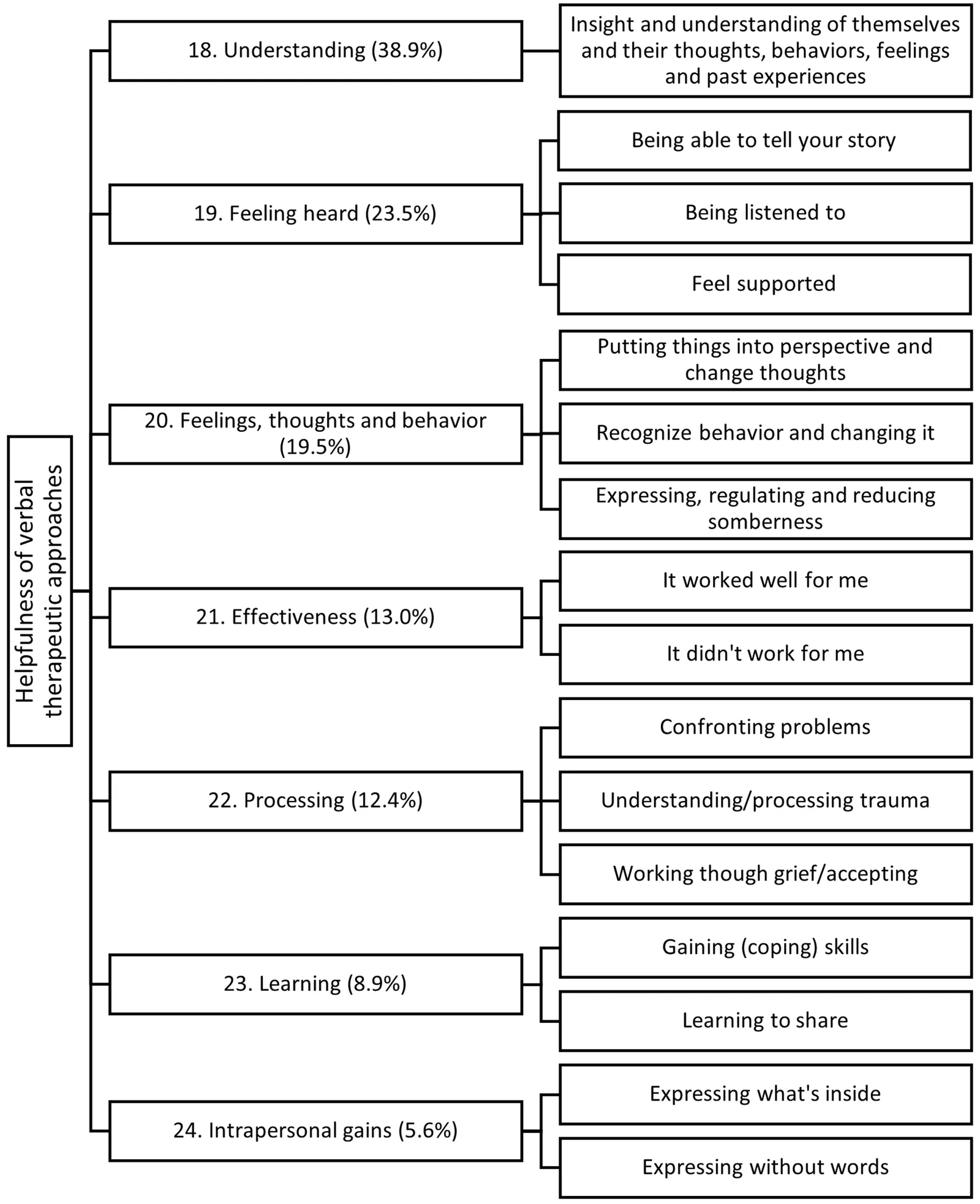
Code tree helpfulness verbal therapeutic approaches. *N_verbal therapies_=486*.

**Figure 4** shows a code tree with the main themes and corresponding codes following the answers of the participants (*n* = 486) on what they experience as helpful about verbal therapeutic approaches.

##### 3.2.3.1 Theme 18: understanding (38.9%)

Many respondents reported that verbal therapeutic approaches helped them gain understanding (38.9%), particularly of their thoughts, feelings, behaviour, and underlying patterns. They described clearer insight into how past experiences related to current symptoms and how to cope with these *“It helped me to figure out what has happened in the past and how it is all connected to each other”.*

##### 3.2.3.2 Theme 19: feeling heard (23.5%)

Verbal therapeutic approaches also supported feeling heard (23.5%) according to participants; being able to share their story, fostered acknowledgement, stability, and security in both the therapeutic relationship and daily life*: “It helps me to feel heard and seen, to normalize things, as encouragement/support to actually take steps, and to regulate emotions when they run very high”.* They were being listened to and felt supported in therapy.

##### 3.2.3.3 Theme 20: feelings, thoughts, behavior (19.5%)

Also, according to the participants, verbal therapeutic approaches contributed to changes in feelings, thoughts and behavior (19.5%), with an emphasis on changes in thoughts by increasing awareness of beliefs. Besides this they mentioned the recognition of behavior and feedback to break behaviour patterns: *“Talk therapy has helped me become aware of my thoughts and to connect them with my reaction to situations”.* Last, they mentioned that they could express there somberness in therapy sessions.

##### 3.2.3.4 Theme 21: effectiveness (13%)

Overall, unprompted, 3.3% of respondents stated that verbal therapeutic approaches always helped them, while 9.7% reported that it had not helped them.

##### 3.2.3.5 Theme 22: processing (12.4%)

The verbal therapeutic approaches further aided processing (12.4%) according to participants including confronting problems, understanding and processing trauma (triggers), working through grief, and using methods such as EMDR *“it helped me to try to let go of the things that happened to me”.*

##### 3.2.3.6 Theme 23: learning (8.9%)

Verbal therapeutic approaches facilitated learning and adapting (8.9%), including gaining coping skills to handle situations differently, and learning to share. *“It gave me tools that I could use myself”.*

##### 3.2.3.7 Theme 24: intrapersonal gains (5.6%)

Finally, verbal therapeutic approaches also supported intrapersonal growth (5.6%), such as an improves self-image (improved self-knowledge, and standing up for oneself more), and a more positive outlook,*. “I got to know myself better”*.

#### 3.2.4 Category 4: unhelpfulness of verbal therapeutic approaches

**Figure 5:**
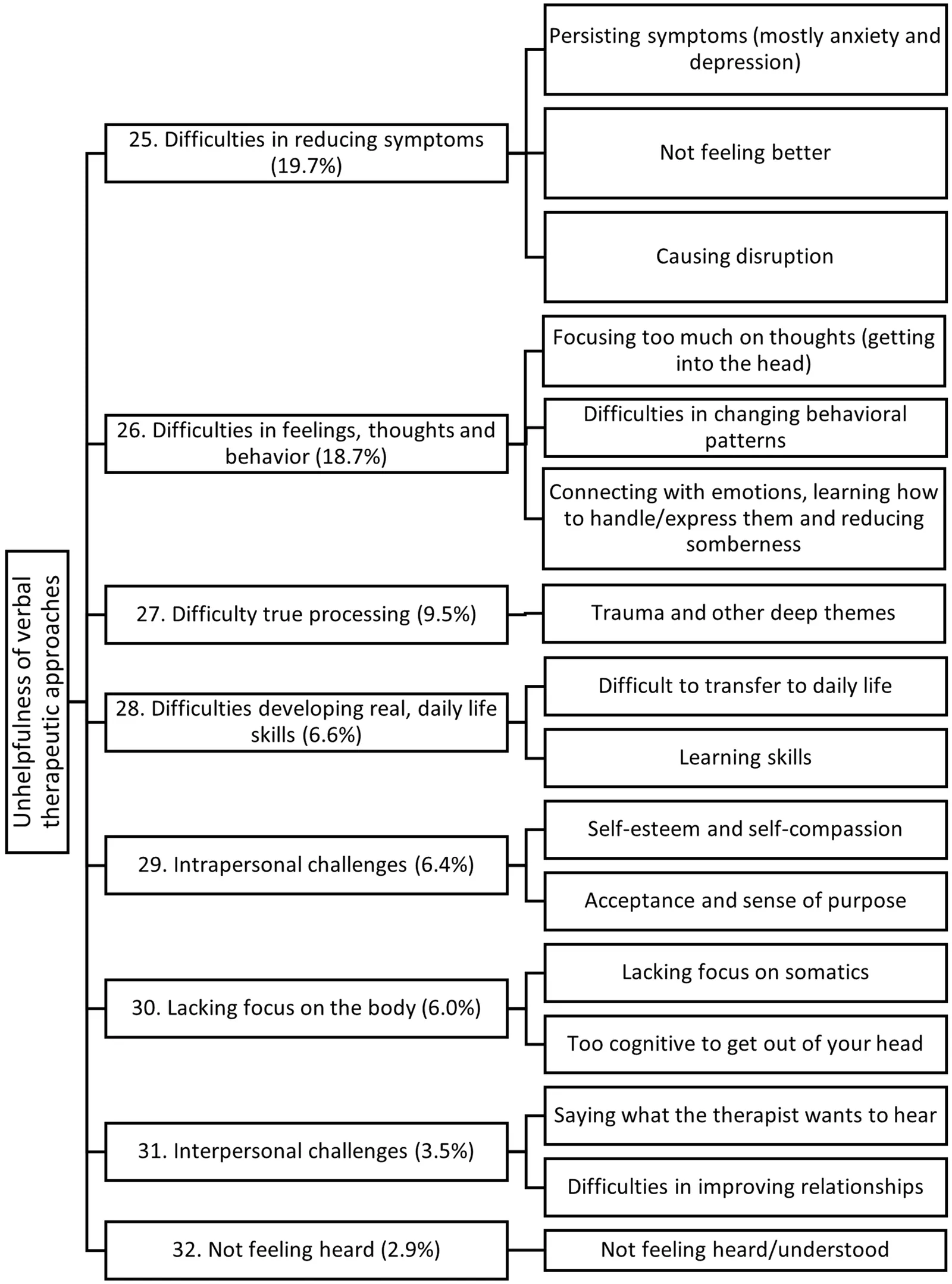
Code tree unhelpfulness verbal therapeutic approaches. *N_verbal therapies_=486*.

**Figure 5** shows a code tree with the main themes and corresponding codes following the answers of the participants (n=486) on what they did not experience as helpful about verbal therapeutic approaches.

##### 3.2.4.1 Theme 25: difficulties in reducing symptoms (19.7%)

A notable number of participants (19.7%) indicated that verbal therapeutic approaches did not significantly reduce symptoms like depression or anxiety, they didn’t feel better, and for some (1.9%) it could evoke disruption. *“Although I have perhaps gained more insight into where my symptoms come from, I do not feel that my symptoms have decreased as a result. In fact, I have started to isolate myself more from the outside world”.*

##### 3.2.4.2 Theme 26: negative feelings, thoughts, behavior (18.7%)

Regarding thoughts, behavior, and feelings, participants mentioned that there was too much focus on cognitions. Verbal therapeutic approaches did not always increase bodily awareness or change dysfunctional patterns for participants. And while 7.2% of them found it helpful to access feelings, 12.8% reported *difficulty* in verbal therapeutic approaches in helping them express or cope with emotions such as sadness, anger, and anxiety. *“I was unable to change my behavior and feelings. There was too much focus on changing thoughts and behavior and too little on my feelings.”*

##### 3.2.4.3 Theme 27: difficulty true processing (9.5%)

Even though participants (12.4%) reported that verbal therapeutic approaches helped with processing trauma, for others (9.5%) it did not address the roots of their problems, leaving trauma symptoms unresolved.

##### 3.2.4.4 Theme 28: difficulties developing real daily life skills (6.6%)

Some (6.6%) highlighted insufficient tools and guidance for developing and applying skills to manage daily life and prevent relapse.

##### 3.2.4.5 Theme 29: intrapersonal challenges (6.4%)

Intrapersonally (6.4%), some participants mentioned that the verbal therapeutic approaches failed to boost their self-esteem or help participants accept symptoms, find purpose, or address existential questions. “*I am still overly perfectionistic and normative towards myself.”*

##### 3.2.4.6 Theme 30: lacking focus on the body (6.0%)

It was noted that verbal therapeutic approaches lacked focus on the body (6.0%) and psychosomatic signals, being too cognitive or “by the book” to be able to get out of your head. *“Talk therapy is often heavily focused on thinking, which meant that with a previous therapist, I kept going around in circles in the same thoughts without any progress in my recovery or without us getting a grip on my symptoms. Consequently, many cognitive techniques/theories such as CBT and schema therapy do not work for me at all. If I could have thought my way out of my problems, I would have done so long ago. I am more than just my thoughts; therefore, in therapy, it is necessary not only to focus on thinking, but also on feeling and doing, and to include the body in that process. Working holistically and from a place of connectedness”.*

##### 3.2.4.7 Theme 31: interpersonal challenges (3.5%)

Interpersonally (3.5%), some participants mentioned that they learned to say what the therapist wanted to hear, or they felt verbal therapeutic approaches did not improve relationships or enhance social skills and boundaries*. “Therapy focuses too much solely on the individual, whereas people are always part of a network such as the family and the social network”*.

##### 3.2.4.8 Theme 32: not feeling heard (2.9%)

Some participants also reported feeling misunderstood or criticized (2.9%) in verbal therapeutic approaches, with too much focus on diagnoses and critiques.

#### 3.2.5 Category 5: difference between verbal therapeutic approaches and CAPTs

**Figure 6:**
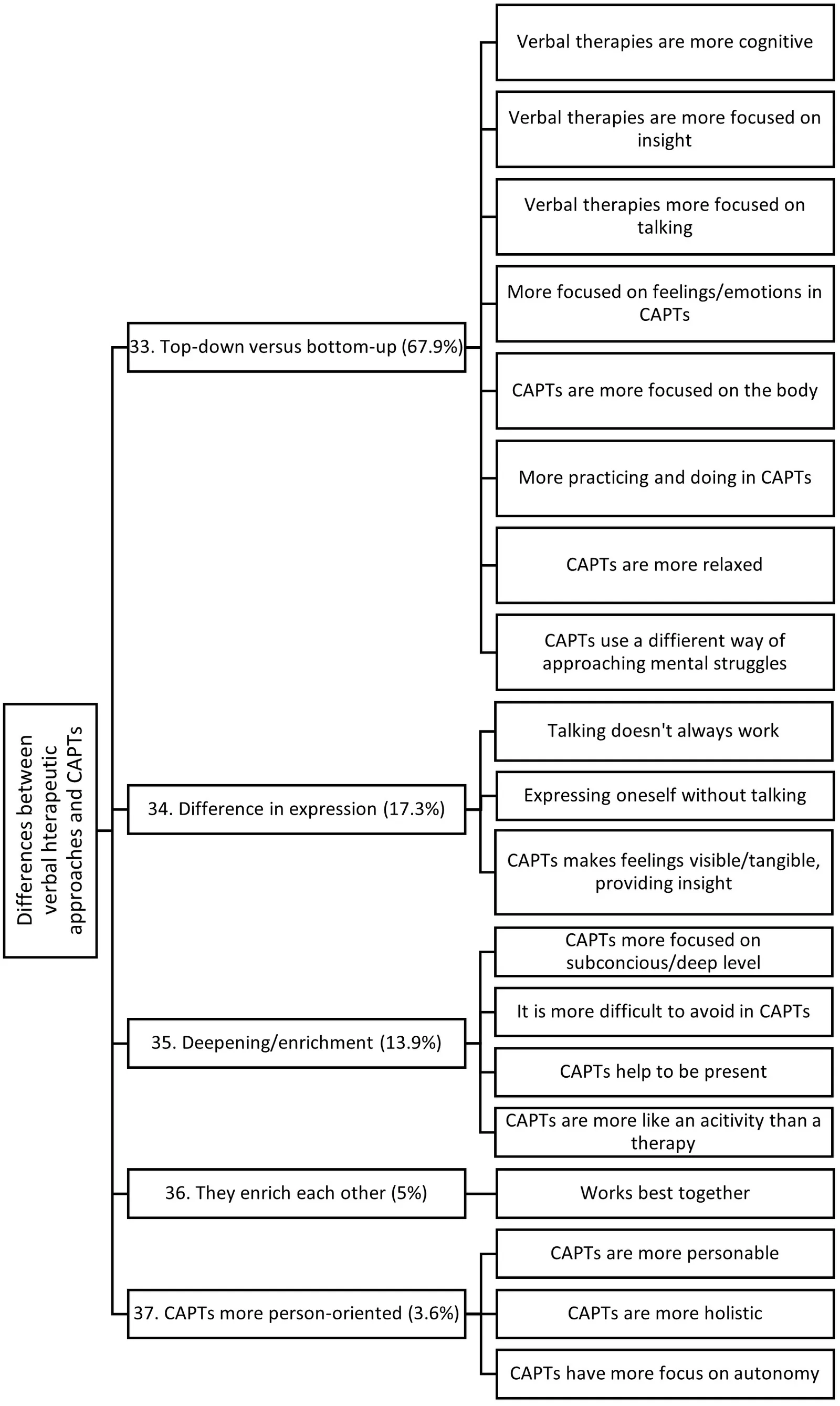
Code tree difference between VTA and CAPTs. *N_CAPTsvsVTA_=330*.

**Figure 6** shows a code tree with the main themes and corresponding codes following the answers of the participants (*n* = 330) on what, in their opinion, is the difference between verbal therapeutic approaches and CAPTs.

##### 3.2.5.1 Theme 33: top-down versus bottom-up (67.9%)

Most mentioned was that verbal therapeutic approaches and CAPTs work in different ways: they are top-town versus bottom-up (67.9%). Participants consistently described verbal therapeutic approaches as more cognitive, insightful and verbal than CAPTs. They characterized it as “*putting puzzle pieces together*”, working mainly with thoughts, explanations, and rational understanding. Verbal therapeutic approaches were further experienced as a place where they could feel heard and tell their story. By contrast, CAPTs were described as more feeling-based and embodied. Participants emphasized that CAPTs are “*more about doing than talking”*, helping them to move out of overthinking and perfectionism. CAPTs provided opportunities for practical rehearsal in small steps according to participants, such as practicing new behaviors, recognizing bodily signals, or setting boundaries. CAPTs were further described to be more relaxed and as “a different way to approach your issues”.

##### 3.2.5.2 Theme 34: difference in expression (17.3%)

Participants further mentioned a different way of expressing in both therapies (17.3%). According to them, talking doesn’t always work and the non-verbal nature of CAPTs allowed expression of emotions and experiences that could not easily be put into words. *“With creative therapy (for me, painting), I can process my emotions in a different way than during the conversations. It flows naturally from within onto the paper. I don’t have to think about it. The therapist helps me express my emotions in a different way”.* Working with images or physical materials within CAPTs was experienced as making inner conflicts tangible and visible, providing insights in a non-verbal way. The tangible products of art therapy (e.g., artworks) served as lasting reminders of insights gained.

##### 3.2.5.3 Theme 35: deepening/enrichment (13.9%)

Related to deepening/enrichment (13.9%), different experiences came forward. Some participants highlighted that CAPTs offered a direct connection to emotions and bodily signals, accessing deeper layers beyond rational thought, and sometimes touching more primitive emotional systems such as the autonomic nervous system. *“Verbal therapeutic approaches appeal to cognition and rationality, while CAPTs speak to other parts of the brain, allowing emotions to surface more easily”.* It was mentioned that it is more difficult be avoidant in CAPTs than in verbal therapeutic approaches: “*With words I could still shut myself off, but with images, drama or movement I couldn’t hide anymore”.* In CAPTs, participants mentioned they could more easily get out of their heads, more relaxed and into present moment. In contrast, some participants experienced CAPTs as primarily accessible or more as an activity than as therapy, mentioning that verbal therapeutic approaches focused more on their actual problems and deeper emotional work. “*Many of the exercises in creative therapy are offered in an accessible way or presented in a playful manner. I missed the applicability to daily life … Sometimes creative therapy is used as an add-on, without having a concrete objective”.*

##### 3.2.5.4 Theme 36: they enrich each other (5%)

5% of participants mentioned that the therapies enrich each other and work best together. *“I think that CAPTs can be a wonderful complement to talk therapy. I would love it if we approached healthcare holistically and looked at what the person seeking help needs at that moment. Working together and determining which form of therapy, with which therapist, can best help that person take the next step”, “CAPTs are very insightful and experiential, and provide input for verbal therapeutic approaches”, “Experiencing things that can serve as input for talk therapy, to discover what patterns are like and what signals exactly mean”.*

##### 3.2.5.5 Theme 37: CAPTs more person-oriented (3.6%)

Finally, 3.6% of participants mentioned CAPTs being more person-oriented: more personal, holistic and more focused on a sense of autonomy.

#### 3.2.6 Code-document analysis

**Table 3** represents the frequencies of codes in each theme. As visible in all code trees, overlapping themes were found in helpfulness and unhelpfulness between verbal therapeutic approaches and CAPTs, although varying in percentages and focus points.

**Table 3:** Total frequencies of codes.

N	Helpfulness CAPTs	Unhelpfulness CAPTs	Helpfulness VTA	Unhelpfulness VTA	Difference CAPTs/VTA
330	543	193			356
486			591	363	

This table represents the amounts of codes for each theme. Noticeable is that the number of codes for helpfulness were much higher than the number of codes for unhelpfulness. VTA, verbal therapeutic approaches.

**Figure 7:**
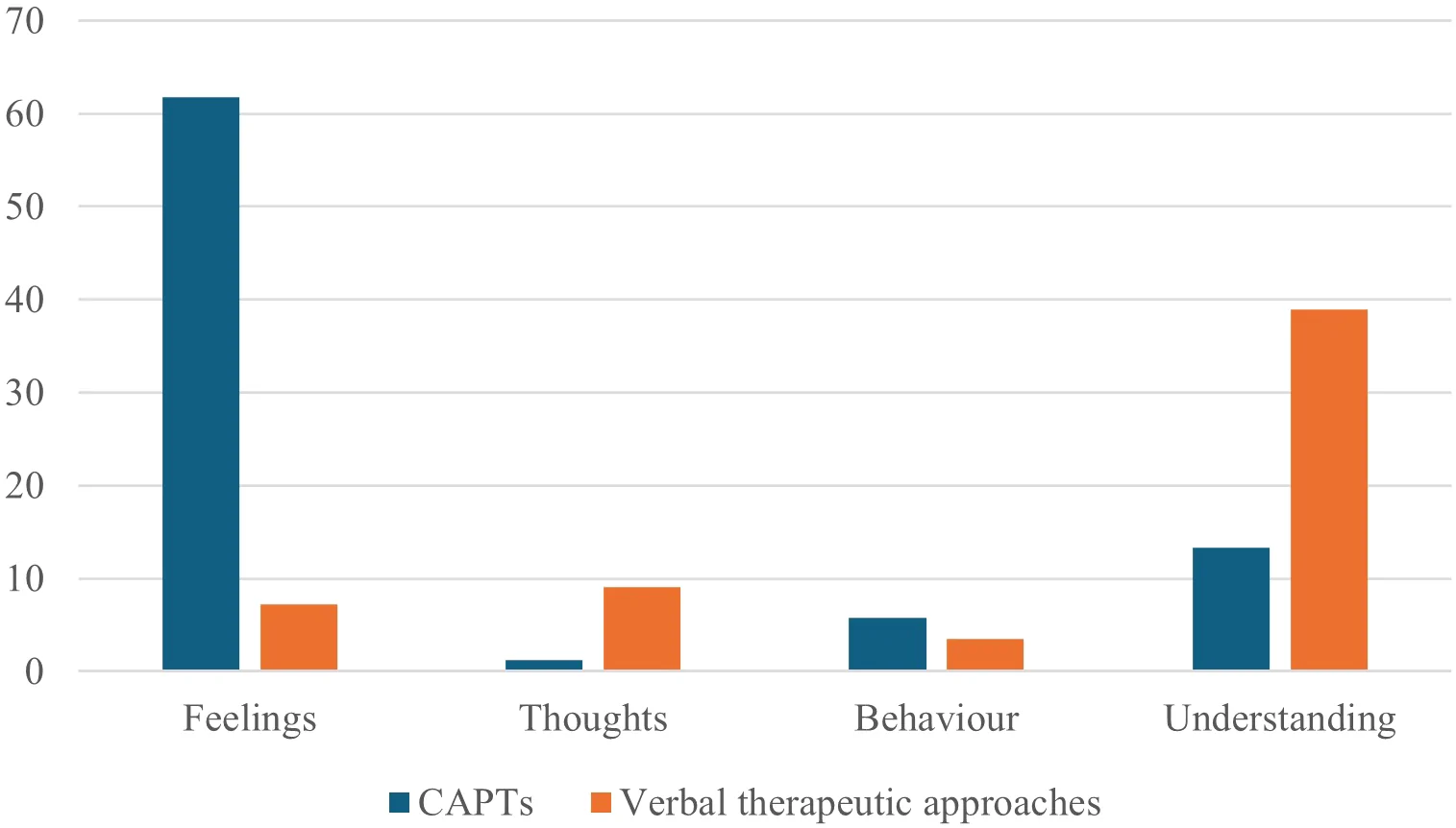
Code-document analysis of concurrent codes on helpfulness. The numbers represent percentages. Codes were divided by number of participants that answered the question (for CAPTs; 330 participants, for verbal therapeutic approaches; 486 participants) x 100. Feelings included both accessing, expressing and regulating emotions and the code ‘feeling and the body’.

**Figure 7** represents the number of times that participants mentioned that CAPTs versus verbal therapeutic approaches helped them with feelings (regulating emotions and feeling in the body), thoughts (understanding them and changing them), behavior (understanding and changing patterns) and understanding (gaining insight into symptoms and unraveling themes).

It is to be noted that CAPTs and verbal therapeutic approaches present a complementary pattern: the helpfulness of verbal therapeutic approaches in insight and understanding and the helpfulness of CAPTs in feeling the body and regulating emotions (see **Figure 7**).

**Figure 8:**
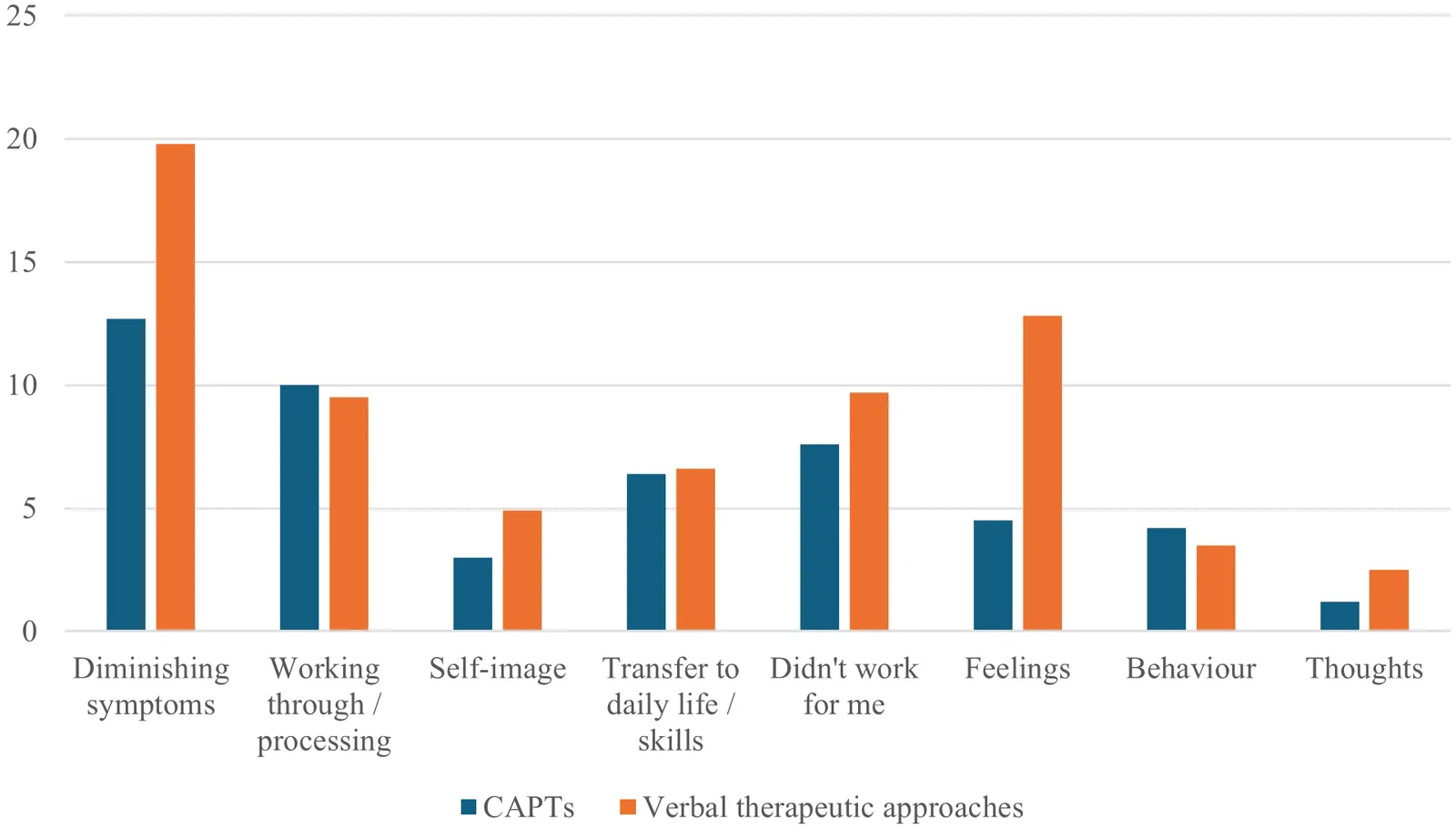
Code-document analysis of concurrent codes on unhelpfulness. The numbers represent percentages. Codes were divided by number of participants that answered the question (for CAPTs; 330 participants, for verbal therapeutic approaches; 486 participants) x 100.

**Figure 8** represents themes in which CAPTs and verbal therapeutic approaches did not help. In all concurring themes except behavior, a higher percentage of participants mentioned that verbal therapeutic approaches didn’t help them. It is notable that it appears to be difficult to really help some patients: to diminish their symptoms, help them to work through trauma and change their deepest self-image and their daily life. For example, 19, 75% of participants mentioned that verbal therapeutic approaches did not help them to diminish their symptoms.

#### 3.2.7 Linguistic inquiry and word count

**Table 4A** provides an overview of the distributions of the absolute total word count per participant. The questions on helpfulness of verbal therapy approaches (*M* = 13, *SD* = 17.45) and unhelpfulness of the verbal therapy approaches (*M* = 10.97 *SD* = 18.42) show a lower mean word count. The questions about helpfulness of the CAPTs (*M* = 17.24, *SD* = 20.52) and unhelpfulness of the CAPTs (*M* = 13.72, *SD* = 20.83) showed a higher mean. Skewness and Kurtosis analyses show that the distributions of all word counts were skewed to the right. 305 participants answered all questions.

**Table 4A:** LIWC table wordcount total descriptives.

	Helpfulness VTA	Unhelpfulness VTA	Helpfulness CAPTs	Unhelpfulness CAPTs
N Valid for comparison	305	305	305	305
Mean	13.00	10.97	17.24	13.72
Median	9.00	5.00	11.00	6.00
Std. Deviation	17.45	18.03	20.52	20.82
Skewness	4.845	5.380	2.714	3.172
Std. Error	0.113	0.113	0.136	0.136
Kurtosis	33.142	43.974	9.136	12.874
Std. Error	0.225	0.225	0.272	0.272
Range	183	207	141	155
Minimum	0	0	0	0
Maximum	183	207	141	155

As noted, percentages of the word counts were compared across the following categories: affect, cognitive processes and its subcategory insight, perception and its subcategory feel, and body.

No significant differences in word count were found for the category affect between responses concerning both the helpfulness and unhelpfulness of CAPTs versus verbal therapeutic approaches (see **Tables 4B**, **4C**).

**Table 4B:** LIWC table helpfulness of verbal therapy approaches versus to helpfulness of CAPTs.

	Helpfulness VTA			Helpfulness CAPTs			Paired sample T-test			Wilcoxon
	M	SD	S.E.	M	SD	S.E.	t	*df*	Sig. (two-sided)	Sig. (two-sided)
Affect	7.83	12.82	.60	7.96	11.30	.63	1.04	304.0	.302	.451
Cognitive	19.64	21.93	1.02	19.33	17.62	.99	.60	304.0	.550	.746
Insight	9.88	17.56	.81	9.23	14.02	.78	.93	304.0	.351	.736
Perception	2.63	6.66	.31	6.29	11.04	.62	-5.66	304.0	.00*	<.001*
Feel	.79	3.24	.15	2.95	8.08	.45	-4.38	304.0	.00*	<.001*
Body	.60	3.13	.15	1.26	4.16	.23	-2.17	304.0	.031*	.011*

LIWC is based on percentages, VTA, Verbal Therapy Approaches. *p <.05.

**Table 4C:** LIWC table unhelpfulness of verbal therapy approaches versus to unhelpfulness of CAPTs.

	Unhelpfulness VTA			Unhelpfulness CAPTs			Paired sample T-test			Wilcoxon
	M	SD	S.E.	M	SD	S.E.	t	*df*	Sig. (two-sided)	Sig. (two-sided)
Affect	9.72	15.89	.74	8.81	16.17	.90	1.04	304.0	.299	.275
Cognitive	15.53	20.06	.93	16.28	20.19	1.13	-.65	304.0	.518	.477
Insight	6.47	14.55	.67	6.15	11.99	.67	.88	304.0	.381	.792
Perception	3.97	12.21	.57	2.92	9.56	.53	1.61	304.0	.108	.059
Feel	2.39	9.61	.45	1.20	5.92	.33	2.31	304.0	.021*	.002*
Body	.79	3.33	.15	.24	1.68	.09	3.20	304.0	.001*	.001*

LIWC is based on percentages, VTA, Verbal Therapy Approaches. *p <.05.

No significant differences in word count were found for the category cognitive processing (including its subcategory insight), between responses concerning both the helpfulness and unhelpfulness of CAPTs versus verbal therapeutic approaches (see **Tables 4B**, **4C**).

For the main category perception, a significant difference in word count was observed for responses concerning helpfulness. Participants used less perception-related words when describing verbal therapeutic approaches (*M* = 2.63, *SD* = 6.66), than CAPTs (*M* = 6.29, *SD* = 11.04), t (304) = -5.67; *p* <.001). No significant difference was found for perception-related word use in responses concerning unhelpfulness.

For its subcategory feel, significant differences were found in both comparisons. Feel-related words were used less frequently in descriptions of the helpfulness of verbal therapeutic approaches (*M* = .79, *SD* = 3.24) than of CAPTs (*M* = 2.95, *SD* = 8.08), t (304) = -4.38, *p* = <.001. Conversely, these words were used more frequently in descriptions of the unhelpfulness of verbal therapeutic approaches (*M* = 2.39, *SD* = 9.61) than of CAPTs (*M* = 1.20, *SD* = 5.92), t (304) = 2.31, *p* = .002.

For the category body, the same pattern emerged: significant differences were found in both comparisons. Body-related words were used less frequently in descriptions of the helpfulness of verbal therapeutic approaches (*M* = .60, *SD* = 3.13) than of CAPTs (*M* = 1.26, *SD* = 4.16), t (304) = -2.17, *p* = .011. Conversely, these words were used more frequently in descriptions of the unhelpfulness of verbal therapeutic approaches (*M* = .79, *SD* = 3.33) than of CAPTs (*M* = .24, *SD* = 1.68), t (304) = 3.20, *p* = .001.

These findings indicate that CAPTs are more frequently associated with bodily and feeling-related words, whereas verbal therapeutic approaches are more often described as lacking in these domains.

#### 3.2.8 Integration of results

The findings highlight the complementary roles of CAPTs and verbal therapeutic approaches. The most-mentioned priority in therapy themes in the closed-ended responses was ‘feeling signals in the body’ (47%), which coincided with the most frequently mentioned helpful aspect of CAPTs (32.4%). ‘Coping with emotions’ was also prominent in the mentioned priorities in closed-ended responses (40%), coinciding with 29% of participants who experienced emotional gains through CAPTs. This emphasizes the value of CAPTs for participants in accessing, expressing, and regulating feelings beyond verbal expression. These findings were corroborated by the LIWC, showing a significant difference in word counts related to ‘feeling’ and ‘body’: mentioned both more in helpfulness of CAPTs and less in *un*helpfulness of CAPTs. In contrast, verbal therapeutic approaches were primarily valued for fostering understanding and insight, with 38.9% of participants reporting gains in awareness of thoughts, behaviors, and relational patterns, even though the LIWC did not show significant differences in word count in cognitions between CAPTs and verbal therapeutic approaches. Both approaches shared overlapping benefits, including behavioral change, trauma processing, and intrapersonal growth, but differed in focus: CAPTs were experiential and embodied, while verbal therapeutic approaches were cognitive and analytical. Despite these benefits, some participants reported persistent anxious or depressed symptoms or limited change in behavior with either approach (e.g., 19.8% said verbal therapeutic approaches did not diminish symptoms; 12.7% said CAPTs did not), highlighting that neither modality alone fully meets all needs. This was corroborated by the insignificant difference in ‘affect’ related words between the modalities. Many participants noted that combining both therapies (insight from verbal therapeutic approaches and experiential engagement from CAPTs), using them complementary to each other, was perceived as valuable.

## 4 Discussion

This study explored peoples lived experiences of creative arts and psychomotor therapies (CAPTs) compared to verbal therapeutic approaches through a qualitative survey of 543 panel members from the Dutch MIND mental health organization (response rate ~14% from 4, 000 invitees).

Participants (*n* = 486 for verbal; *n* = 330 for CAPTs) described CAPTs and verbal therapies as complementary rather than substitutive. Specifically, 67.9% highlighted CAPTs’ bottom-up, embodied focus (“feeling” and “experiencing” over “talking”) versus verbal therapies’ top-down cognition, aligning with theoretical distinctions (26).This perception supports integrating modalities to address diverse needs, as 5% explicitly endorsed combinations for holistic care.

CAPTs were frequently endorsed for improving emotional contact (35.8%), bodily awareness (32.4%), and experiential learning (20.3%). These map onto established mechanisms: embodiment (“out of the head” into body signals), emotional eliciting, regulation and processing, concretization (visualizing/materializing abstracts), and symbolism (non-verbal expression), consistent with prior models (5, 16). LIWC analyses supported this, with significantly higher “feel” (*p* <.001), “perception” (*p* <.001), and “body” (*p* = .011) word use for CAPTs’ helpfulness than for verbal therapeutic approaches. No novel mechanisms emerged, but participants extended and corroborated existing theories.

Verbal approaches facilitated insight (38.9%), feeling heard (23.5%), and processing (12.4%), but were critiqued for lacking embodiment (12.8% struggled with emotions; 6.0% noted absent body focus). Third wave “talk” therapies such as mindfulness-based cognitive therapy place more significance on the body than earlier verbal therapeutic approaches. However, the incorporation of the body and its signals and feelings remain an underlit theme in first-choice treatment for mental health (27). This was corroborated by the LIWC (lower body words, *p* = .011).

Although most found therapies helpful, 7.6%-19.8% reported unhelpfulness (e.g., persistent symptoms, poor transfer to daily life), the highest being for symptom reduction in verbal and creative arts therapies. This underscores a recovery oriented understanding of mental health, in which “recovery” may not always mean full remission or symptom eradication, but instead may involve learning to live with ongoing vulnerability, developing coping strategies and self-management capacities, resonating with emphasis on subjective recovery over categorical cure (28), tailoring interventions to individual priorities like body signals (top-ranked theme).

Strengths of this study are its sample size (*n* = 543; 63% CAPTs-exposed), exceeding typical qualitative studies (*n* < 100), with rich data from open responses, rigorous inductive thematic analysis (23; triangulation by three coders), and LIWC validation.

Limitations of the study should also be acknowledged. First, respondents were aware that this study was conducted by CAPT therapists and focused on CAPTs, as the survey topic was visible when distributed to panel members. This may have created response bias by attracting individuals with pre-existing interest in CAPTs (either favorable or unfavorable attitudes), potentially influencing both participation and responses. We sought to mitigate this limitation by incorporating feedback from non-CAPT therapists (a methodologist, psychologist, and expert by experience) during study development, and by asking balanced questions about both positive and negative aspects of CAPTs and verbal therapeutic approaches. The response rate was 14%, which may have limited generalizability. Due to the lack of available data on non-respondents, we were unable to conduct a non-response analysis to assess potential response bias. 83% of the responders were women, which may also limit generalizability of the findings to non-women. Second, not all participants had experienced both CAPTs and verbal therapeutic approaches, limiting direct within-person comparison. Third, although the open-ended questions yielded thematically rich responses across participants, their brevity may have constrained the depth and nuance of individual accounts. The questions were designed to capture participants’ immediate reflections on therapy, and the thematic analysis benefited from a large number of responses, allowing for robust pattern identification at the group level. However, the format may have limited opportunities for participants to elaborate on complex, ambivalent, or context-dependent experiences, potentially leading to an emphasis on more immediately salient aspects. As a result, less salient or more nuanced perspectives may be underrepresented compared to what might be obtained through longer instruments or in-depth interviews. Fourth, there are many types of verbal therapeutic approaches and CAPTs, which have been generalized in this study. Last, our sample is drawn from a national mental health patient panel in the Netherlands with mainly high educated female respondents; representation and generalizability to other cultural or clinical contexts may therefore be limited.

These patient-informed mechanisms challenge most guidelines prioritizing verbal therapies as first-line care, where CAPTs remain underrepresented despite growing evidence (29). By valuing service user perspectives (the third EBP pillar (18)_this study hopes to aid in shared decision-making, potentially reducing attrition (21, 30). Our findings reinforce the argument that experiential knowledge should count alongside clinical evidence and adds to studies on working mechanisms that have expanded in CAPTs in recent years (5, 16, 31).

The following clinical considerations are intended as hypothesis-generating suggestions derived from qualitative findings and warrant further empirical validation. Consistent with the perspectives of people with lived experience, our findings suggest that mental health services could consider embedding creative arts and psychomotor therapies (CAPTs) within a coordinated, multidisciplinary treatment offer alongside verbal therapeutic approaches, rather than positioning them solely as secondary options. This recommendation is grounded in participants’ endorsement of complementarity (67.9%), reflecting a distinction between bottom-up, embodied processes and top-down cognitive-reflective work. Concretely, the identified themes point to several possible actionable strategies. Given the prominence of bodily awareness, emotional contact, and emotion regulation opportunities, services could incorporate brief screening at intake for somatic symptoms (50%), trauma (42%), and emotion regulation needs, using this information to guide treatment allocation. For individuals who primarily struggle to access or regulate emotions verbally, CAPTs may be considered early in treatment as a first-line or preparatory modality, particularly in light of themes such as body-signal awareness (47%) and emotional elicitation (35.8%). In addition, the observed complementarity supports deliberate sequencing or parallel provision of CAPTs next to verbal therapy approaches, in which CAPTs facilitate concretization, embodiment, and emotional access, which can subsequently be processed in verbal therapy to support insight (38.9%) and transfer to daily life. To operationalize this, interdisciplinary collaboration, such as shared treatment planning and regular case consultation between CAPT and verbal therapists, may help align modalities around shared goals while preserving their distinct mechanisms of action. These practice-oriented implications are derived from service user perspectives and warrant further empirical validation, particularly in studies examining feasibility, effectiveness, and optimal sequencing of multimodal interventions. For research, we recommend building on to this patient-informed knowledge by conducting pragmatic trials that integrate service user perspectives into study design, such as co-developing outcome measures for body awareness and emotion regulation alongside traditional endpoints. These could include moderator analyses (e.g., baseline somatic, symptom severity, treatment length or trauma symptoms) and longitudinal tracking of real-world transfer, while prioritizing diverse samples to improve generalizability beyond this Dutch, female-dominated panel. Such approaches would honor the value of lived experience as a core pillar of evidence-based practice, bridging experiential insights with rigorous evaluation.

In sum, in this Dutch sample, service users perceive CAPTs and verbal therapies as complementary via distinct mechanisms. By integrating both experiential, embodied methods of the CAPTs and reflective, verbal techniques of verbal therapeutic approaches, mental health services may better address the diverse ways people heal and recover. These findings reflect the Dutch context, but the complementary mechanisms identified could have broader relevance beyond the Netherlands, warranting further research in other countries. However, the absence of data on non-respondents precluded a non-response analysis, and potential response bias should therefore be considered when interpreting the generalizability of these results.

## Data Availability

The raw data supporting the conclusions of this article will be made available by the authors, without undue reservation.
