# Supplementary material for: Feeling versus understanding: lived-experience insights into perceived mechanisms of change of creative arts and psychomotor therapies versus verbal therapeutic approaches
Source: Front Psychiatry. 2026 Aug 27;17:1863648. doi: 10.3389/fpsyt.2026.1863648 (PMC13559429; doi:10.3389/fpsyt.2026.1863648)
Supplement: Supplementary file 1 [file Supplementaryfile1.docx]

**Supplementary material**

**Supplement 1: Full survey**

**Part 1: Background Information**

The questionnaire begins with a few questions about background information.

1. **My age** [numbers only]
2. **I am...**
   A man 
   A woman 
   Other
3. **What is your highest completed level of education?**
   (Select one answer) 
   0 Primary school 
   1 Lower vocational education (e.g., domestic science school, LTS, LHNO, LEAO) 
   2 MULO, MAVO, 3 years of HAVO 
   3 Higher general secondary education (e.g., completed HAVO) 
   4 Preparatory scientific education (HBS, VWO) 
   5 Intermediate vocational education (e.g., MTS, MEAO, INAS, VHBO) 
   6 Higher professional education (HBO) 
   7 University education (WO) 
   8 Other, namely: ………………………………………………………

**Part 2: Complaints and Treatment**

**4. Have you experienced psychological complaints in the past 3 months?** 
(Select one answer) 
0 Yes 
1 No, I am filling out this questionnaire for a family member 
1 No, I am filling out this questionnaire for a friend 
1 No, another reason

**5. What psychological complaints have you experienced?** 
Some people prefer to mention a diagnosis, others prefer to describe the complaints they experienced. 
(You can select multiple answers)

- Difficulty controlling my emotions
- Negative thoughts about myself
- Difficulty with relationships with others
- Not really knowing who I am
- Mood swings
- Difficulty controlling my behavior
- Anxiety disorder
- Mood disorder
- Addiction
- Trauma
- Psychosis
- Personality disorder
- Open answer

**6. How would you rate your overall quality of life over the past week?** 
By “quality of life” we mean an overall sense of satisfaction — having a pleasant and meaningful life.

1 2 3 4 5 6 7 
Very poor                     	Very good

**7. Are you currently receiving treatment for your psychological problems?** 
(Multiple answers possible)

- Yes, with talk therapy (psychotherapy)
- Yes, with arts therapies — visual art, music, drama, dance, play, or psychomotor therapy (PMT)
- Yes, with medication
- No, treatment has been completed
- No, I stopped treatment (it didn’t work or I didn’t like it)

**8. In what ways has talk therapy helped you?** 
**9. In what ways has talk therapy not helped you?**

**Part 3: Arts Therapies**

**1) I have experience with arts therapies** (such as art therapy, dance therapy, drama therapy, music therapy, or psychomotor therapy). 
[Short explanation of arts therapies; source: [www.vaktherapie.nl](http://www.vaktherapie.nl/), simplified] 
Arts therapy is a practical and experience-based form of treatment used in mental health care (GGZ). It may include creative approaches such as painting and clay modeling (art therapy), music, theater (drama), dance, and movement or sports (psychomotor therapy). Through these methods, people can recognize and work on their ways of acting and feeling from daily life. In arts therapy, the focus is not on talking, but on doing and experiencing.

Yes → If yes, go to question 1A 
No → If no, go to Part 4

**1A) I am familiar with the following forms of arts therapy** (multiple answers possible)

- Art therapy
- Dance therapy
- Drama therapy
- Music therapy
- Psychomotor therapy
- Play therapy

**2. The following statements are about arts therapies. Please indicate to what extent you agree.** 
(5-point Likert scale: totally true – true – neutral – not true – totally untrue)

- Combining multiple forms of arts therapy in a module has added value compared to just one form.
- I think arts therapy complements and/or adds value to talk therapy.
- Arts therapy helps in sensing bodily signals.
- Arts therapy makes intangible themes (such as emotions) clearer, which helps in changing them.
- In arts therapy, I can symbolically express emotions and important personal themes.
- Arts therapy provides insight into emotions (my own and others’).
- Arts therapy is a good place to explore and recognize my emotions.
- Arts therapy is a good place to work on (negative) thoughts.
- Exercises in arts therapy help to change behavior.
- Arts therapy provides a safe environment to practice new behavior.
- Behavior learned in arts therapy can be applied in daily life.
- After arts therapy, I can better express my emotions and themes in words.

**Open questions about arts therapy:**

- What do you think is the difference between arts therapy and talk therapy?
- In what ways has arts therapy helped you?
- In what ways has arts therapy not helped you?

**Part 4: Questions About Important Themes in Recovery**

We aim to develop a therapy that helps clients deal with large and small challenges in daily life. Because no therapy can solve all challenges, we would like to focus on what clients and relatives find most important. Which of the following challenges in daily life do you think are the most important to include in therapy? You may choose up to 5 topics.

- Reducing symptoms
- Strengthening helpful skills
- Noticing physical signals accurately
- Learning to adapt to changing circumstances
- Developing practical skills
- Gaining insight into your own behavior
- Better understanding the behavior of others
- Recognizing your emotions
- Managing your emotions
- Knowing your strengths
- Knowing who you are
- Knowing what others want
- Knowing what you want
- Learning to balance your own wishes with those of others
- Being able to enjoy things
- Speaking to yourself in a positive way
- Fulfilling your own needs in a healthy way
- Changing (negative) thoughts
- Gaining new, positive experiences
- Developing healthy ways of relating to yourself
- Developing healthy ways of relating to others
- Processing the past

**2) Based on this survey, we aim to develop an arts therapy program that includes different forms and goals to help reduce symptoms and strengthen mental health (resilience). Which activities and goals do you think fit best? Please choose up to 5.**

- Increasing spontaneity and playfulness
- Practicing new behavior
- Representing a problem in another (symbolic) form
- Increasing flexibility by practicing spontaneous reactions (improvisation)
- Expressing one’s own feelings (self-expression)
- Becoming kinder to yourself (self-compassion)
- Increasing creativity
- Improving cooperation
- Enhancing body awareness
- Setting boundaries
- Working on acceptance
- Learning to give direction to life based on what matters to you
- Expressing and managing emotions
- Getting to know yourself through experimentation
- Connecting with your own emotions and desires
- Connecting with the emotions and desires of others
- Learning to recognize bodily signals (body awareness)
- Increasing self-confidence
- Increasing self-control
- Learning to distinguish between what belongs to you and what belongs to others
- Learning to respond appropriately to bodily signals
- Working with a timeline to represent your life story

**Part 5: Questions About Treatment of Personality Disorders and Schema Therapy**

A **diagnosis of a personality disorder** is often associated with:

- Problems with one’s own emotions
- Expressing and regulating emotions
- Negative self-image
- Difficulty regulating one’s behavior
- Problems in relationships with others

The following questions concern treatment for personality disorders.

**I have had (or currently have) treatment for a personality disorder.** 
Yes 
No 
Yes, for the relative I am completing this for 
No, for the relative I am completing this for

**I have experience with schema therapy.** 
[Short explanation of schema therapy; source: [www.schematherapie.nl](http://www.vaktherapie.nl/), simplified] 
Schema therapy is a form of psychotherapy for people with personality problems or other long-term difficulties, such as recurring depression. Schema therapy helps you recognize and change unhealthy patterns (schemas and modes) so you can think, feel, and act differently. You learn to sense your needs and respond to them in a healthier way (the “healthy adult” mode). This helps you to give direction to your life and improve your relationships with others.

Yes → If yes, go to question 2A 
No → If no, go to the end

**2A) Below are several statements about your experience with schema therapy. Please rank them in order of importance to you.** 
(1 = Most important, 6 = Least important)

- Strengthening the healthy adult (learning to sense and respond to your needs in a healthier way)
- Strengthening the happy child (experiencing more connection, freedom, and spontaneity)
- Reducing unhealthy modes
- Changing my thoughts (e.g., challenging unhealthy beliefs/schemas)
- Changing my behavior (e.g., through behavioral experiments)
- Changing my feelings (e.g., using chair work and imagery exercises)
