# Supplementary material for: Feeling versus understanding: lived-experience insights into perceived mechanisms of change of creative arts and psychomotor therapies versus verbal therapeutic approaches
Source: Front Psychiatry. 2026 Aug 27;17:1863648. doi: 10.3389/fpsyt.2026.1863648 (PMC13559429; doi:10.3389/fpsyt.2026.1863648)
Supplement: Supplementary file 2 [file Supplementaryfile2.docx]

**Supplementary material**

**Supplement 2: “Other/remaining” theme summary**

The respondent emphasizes that **experienced and skilled therapists are essential** for any form of therapy to be effective. Talk therapy with a downtoearth, realistic approach was personally the most effective. However, therapy outcomes often depended strongly on **the therapist’s attitude, experience, and genuine interest**. When therapists didn’t listen attentively or treated clients impersonally (“just another number on the waiting list”), therapy failed.

Experiences with **psychomotor therapy (PMT)** were mixed: sometimes ineffective or even counterproductive due to therapists who lacked understanding or empathy. Respondents noted that **art or bodyoriented therapies** were not always integrated or respected within talk therapy, which limited their potential benefit. Better coordination between different therapy forms improved results.

Several participants argued that **therapy focuses too narrowly on the individual**, ignoring broader social systems like family, culture, and community. They called for a more **holistic, systemic approach**, including societal responsibility for creating a healthier environment.

Other recurring themes:

- Group therapy can be helpful for social learning but often feels unsafe for trauma survivors.
- There are significant **barriers to access**: limited sessions, high costs, reimbursement issues, and treatment caps.
- **Continuity and matching** of therapy type to each client’s needs at the right time is crucial.
- Some found CAPTs beneficial, especially when aligned with personal inclinations (e.g., art, music), but **choice and autonomy** were vital for success.
- Many stressed the importance of **diagnostic accuracy**, the **therapeutic relationship**, and **a safe, judgment free environment**.
- Frustration arose around **fragmented care** and **bureaucratic health system limitations**.

Finally, one participant critiqued the emphasis on always turning experiences into something “positive,” noting that **healing sometimes requires space to feel negative emotions first** before transformation can occur.
